# Advances in CRISPR/Cas9-Based Gene Editing in Filamentous Fungi

**DOI:** 10.3390/jof11050350

**Published:** 2025-05-01

**Authors:** Bin Ma, Yimiao Li, Tinghui Wang, Dongming Li, Shuang Jia

**Affiliations:** School of Life Sciences, Inner Mongolia University, Hohhot 010070, China; m_monologue@163.com (B.M.); 22008046@mail.imu.edu.cn (Y.L.); 15933261582@163.com (T.W.); lidongming@lzu.edu.cn (D.L.)

**Keywords:** CRISPR/Cas9, filamentous fungi, genome editing

## Abstract

As an important class of microorganisms, filamentous fungi have crucial roles in protein secretion, secondary metabolite production and environmental pollution control. However, characteristics such as apical growth, heterokaryon, low homologous recombination (HR) efficiency and the scarcity of genetic markers mean that the application of traditional gene editing technology in filamentous fungi faces great challenges. The introduction of the RNA-mediated CRISPR/Cas (clustered regularly interspaced short palindromic repeat/CRlSPR-associated protein) system in filamentous fungi in recent years has revolutionized gene editing in filamentous fungi. In addition, the continuously expressed CRISPR system has significantly improved the editing efficiency, while the optimized sgRNA design and reduced cas9 concentration have effectively reduced the off-target effect, further enhancing the safety and reliability of the technology. In this review, we systematically analyze the molecular mechanism and regulatory factors of CRISPR/Cas9, focus on the optimization of its expression system and the improvement of the transformation efficiency in filamentous fungi, and reveal the core regulatory roles of HR and non-homologous end-joining (NHEJ) pathways in gene editing. Based on the analysis of various filamentous fungi applications, this review reveals the outstanding advantages of CRISPR/Cas9 in the enhancement of protein secretion, addresses the reconstruction of secondary metabolic pathways and pollutant degradation in the past decade, and provides a theoretical basis and practical guidance for the optimization of the technology and engineering applications.

## 1. Introduction

Fungi, as a unique group of eukaryotes, exert a dual strategy of asexual and sexual reproduction in their life cycle. In asexual reproduction, spores form mycelial networks through germination and rapidly spread to form clonal populations, while sexual reproduction produces genetically diverse spores through mycelial fusion, nuclear matings and meiosis, a dynamic process that is critical in ecological adaptation and evolution [1]. Fungi are extremely diverse, ranging from unicellular yeasts, such as *Saccharomyces cerevisiae*, to filamentous forms, such as *Aspergillus*, and large ascomycetes such as *mushrooms* in the family Stramenophora. Fungi show remarkable heterogeneity in their metabolic capacity, from saprophytes that decompose lignocellulose to species that synthesize antibiotics, toxins, or medicinal secondary metabolites, and to taxa that form symbiotic mycorrhizas with plants [1,2]. It is thought that the number of fungal species worldwide may exceed one million, but only about 150,000 have been described, and their untapped genetic, metabolic, and applied potential provides a vast resource for the integration of biotechnology [3].

Filamentous fungi, as a class of saprophytic eukaryotic microorganisms widely distributed in various ecological environments, have had a profound impact on human society. With the development of industrialization and the modernization of agriculture and medicine, there is a growing demand for the exploitation and utilization of these organisms. Filamentous fungi play the role of decomposers, participate in material cycles and metabolic processes, and are pivotal in the global carbon cycle, maintaining ecological balance through the degradation of organic matter [4,5]. In addition, filamentous fungi, as a cost-effective ‘biofactory’ capable of synthesizing a diverse range of metabolites and industrial enzymes on a large scale, show extensive potential applications in a variety of fields such as bioleaching, bioremediation, and soil fertility enhancement [6]. In terms of heterologous protein expression, antibiotic and organic acid production [7], filamentous fungi are particularly prominently used, and the diversity of substances they produce and their wide range of applications have made them a hotspot of industrial microbiological research. However, certain filamentous fungi such as *Aspergillus* and *Fusarium* are capable of producing highly carcinogenic and mutagenic toxins [8], posing a serious threat to plant, animal and even human health. For example, rice blast disease caused by *Rhizopus oryzae* causes serious damage to rice crops, leading to severe economic losses. Therefore, in-depth research on filamentous fungi not only helps to explore their potential for industrial and pharmaceutical applications, but is also essential for preventing and controlling their negative effects [9].

In the study of the biological properties of filamentous fungi, the effectiveness of genetic engineering manipulation is the key to research progress. With the rapid discovery of new species of filamentous fungi and the increasing potential of their applications, the development of genome-editing strategies to improve strains and increase the variety and yield of metabolites has become an urgent task in the field of scientific research. The rapid development of modern gene-editing technologies has provided researchers with a variety of molecular biology tools, including homologous recombination (HR) and CRISPR/Cas genome-editing technologies (clustered regularly interspaced short palindromic repeats/CRISPR-associated protein), among others. The innovation and application of these technologies in the field of fungal gene editing have greatly contributed to the development and improvement of strains [10]. CRISPR/Cas9 technology, as a highly developed genome-editing tool, has demonstrated great potential in tagging, regulating, modifying, editing, and localizing genomic loci in a wide range of cells and organisms due to its high efficiency, convenience, simplicity of construction, ease of manipulation, cost-effectiveness, potential for highly selective targeted mutagenesis, and ability to edit genes in most regions.

The CRISPR/Cas9 system originates from the adaptive immune mechanism of bacteria and archaea, and its gene-editing function relies on the synergistic action of the Cas9 nuclease and single-stranded guide RNA (sgRNA). The Cas9 protein interacts with the sgRNA to direct endonuclease activity to the proximal end of the protospacer adjacent motif (PAM) sequence. PAM sequences are two- to six-nucleotide sequences located downstream of the target DNA sequence and are essential for the recognition of the Cas9 protein [8,11,12]. The custom-designed sgRNA recognizes its target sequence and allows Cas9 endonuclease to cleave the sense strand 3 base pairs (bp) and antisense strand, 3 bp upstream of the PAM sequence (NGG), forming a double-strand break (DSB). Host cells respond to DSBs through two major repair pathways: non-homologous end-joining (NHEJ), which is prone to knockout or shifting mutations, and homology-directed repair (HDR), which relies on exogenous donor templates to achieve precise knock-in or replacement. This core mechanism of “recognition–cleavage–repair” grants CRISPR/Cas9 high efficiency and programmability in genome editing, but its actual efficiency is significantly affected by the design of sgRNAs, the compatibility of PAMs, and the repair pathways of the host (e.g., the predominance of NHEJ in filamentous fungi), and needs to be optimized by delivery systems (e.g., RNP complexes) [13].

Since the first in vitro programming of CRISPR/Cas9 by Jennifer Doudna and Emmanuelle Charpentier in 2012, the technology has rapidly become a versatile tool for gene editing across species [14]. It has been applied to simplify and metabolically engineer the genome of *Escherichia coli* in the early days of prokaryotes; to achieve efficient editing in model eukaryotes such as *Saccharomyces cerevisiae*, zebrafish, mouse, and *Arabidopsis thaliana* to advance functional genomics; to achieve multi-gene editing in mammalian cells (e.g., HEK293); and to improve disease/resistance traits in agricultural crops (rice, wheat). For eukaryotes with complex cell walls or multiple nuclei (e.g., filamentous fungi, algae), delivery systems and repair mechanisms need to be optimized [15,16,17,18,19]. Despite the wide range of applications, editing efficiency is significantly affected by species-specific factors (e.g., DNA repair preference, sgRNA design compatibility). For example, mammalian cells rely on HDR repair, whereas NHEJ dominates in plants and fungi, requiring the targeted design of donor templates or the inhibition of the NHEJ pathway to improve precision editing rates [15,20]. In 2013, DiCarlo et al. successfully applied CRISPR/Cas9 for the first time in *Saccharomyces cerevisiae*, but yeast as a unicellular fungus is less difficult to edit [21]. In 2015, Nødvig et al. developed a plasmid-based Cas9/sgRNA co-expression system in *A. nidulans*, marking a breakthrough in the CRISPR editing of filamentous fungi for efficient gene knockout [22].

The aim of this review is to introduce the basic structure, classification, mechanism of action, and influencing factors of CRISPR/Cas9 genome-editing technology, and to analyze its effects on filamentous fungi. In addition, the review will explore the expression and transformation mechanisms of CRISPR/Cas9 in filamentous fungi, as well as the regulatory roles of HR and NHEJ pathways in their gene editing. Finally, this review will summarize advances in CRISPR/Cas9 genome editing across diverse filamentous fungi and provide theoretical and practical guidance for optimizing its application in fungal genomics.

## 2. Brief About CRISPR/Cas9

**Figure 1:**
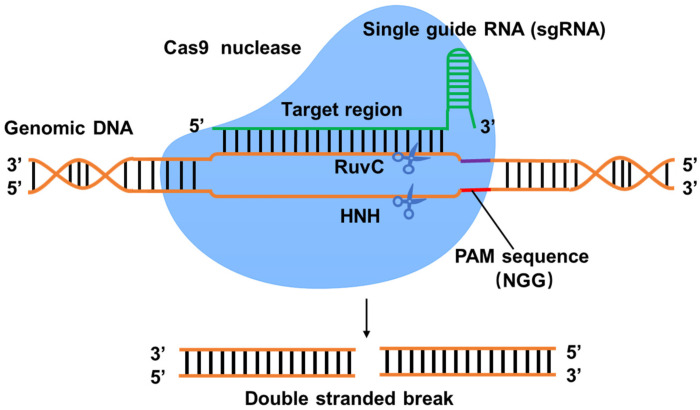
Schematic diagram of the CRISPR/Cas9 system. The Cas9 protein interacts with the single-guide RNA (sgRNA) to direct endonuclease activity to the proximal end of the protospacer adjacent motif (PAM) sequence. The custom-designed sgRNAs recognizes its target sequence and allows Cas9 endonuclease to cleave the sense strand 3 base pairs (bp) and antisense strand 3 bp upstream of the PAM sequence (NGG). The binding of sgRNAs to the target sites induces Cas9 endonuclease to create a double-strand break (blunt end) on the genomic target.

The CRISPR/Cas system is a bacterial and archaeal defense mechanism against phage invasion and foreign genetic material [23,24]. The core components of the system include the leader sequence, the Cas protein, and the CRISPR array. Bacterial genomes typically contain one to two CRISPR/Cas motifs. The leader sequences are between 20 and 534 base pairs in length, are rich in adenine (A) and thymine (T), and contain promoter elements, usually localized at the 5′ end of the repeat spacer sequence. The Cas protein, a double-strand DNA nuclease, consists of two key structural domains, HNH and RuvC [25], which are responsible for cleaving DNA strands complementary to CRISPR RNA (crRNA) and non-complementary strands, respectively, thus triggering double-stranded DNA breaks (DSBs) (Figure 1) [26]. CRISPR arrays, on the other hand, consist of a series of discontinuous repeats and spacers of similar length, which are arranged in a specific order and together form the clustered CRISPR gene structure.

The development of CRISPR/Cas technology has benefited from diverse combinations of Cas genes, leading to multiple CRISPR/Cas systems classified into two classes and six subtypes (types I–VI) [13,27]. Class I systems are more complex, containing multiple nuclease subunits covering types I, III, and IV, which are found in both bacteria and archaea and whose effector complexes consist of four to seven Cas protein subunits. The second class of systems is relatively simple, including types II, V, and VI, which are mainly found in bacteria, and their effector complexes consist of a single multidomain protein [28]. Given the high specificity function and low off-target effect of Cas9 proteins, Cas9 genes have been widely used in CRISPR technology [26]. In particular, the type II CRISPR/Cas9 system derived from *Streptococcus pyogenes* has emerged as a genome-editing tool with unique advantages and a wide range of applications [29,30,31]. The CRISPR/Cas9 system comprises single-guide RNA (sgRNA) and Cas9 endonuclease. Following transcription, sgRNA folds into a tertiary structure, binds to Cas9 to form a ribonucleoprotein complex, which recognizes and guides the Cas9 endonuclease to specific target DNA sequences, and cuts approximately 20 nucleotides upstream of the protospacer adjacent motif (PAM) sequence, generating DSBs (Figure 1) and thereby activating the DNA damage repair mechanism. Gene insertions and deletions are mediated by HR and NHEJ [32].

The adaptive immune response in which the CRISPR/Cas9 system is involved can be divided into three stages. The initial stages involve the integration of an exogenous DNA fragment, which is processed and inserted as a new spacer sequence into the CRISPR gene array. Subsequently, the precursor CRISPR RNA (pre-crRNA) is transcribed and processed to form the mature CRISPR RNA (crRNA), which comprises repeat and spacer sequences. The mature crRNA binds to the Cas9 protein, which in turn forms the CRISPR ribonucleoprotein complex. In the third stage, the ribonucleoprotein complex directs Cas9 to recognize and cleave exogenous nucleic acids via complementary pairing between the crRNA spacer sequence and target DNA [27,33].

The CRISPR/Cas9 system is valued for its ability to introduce multiple single-directed RNAs (sgRNAs), its multi-gene editing and low toxicity in cells. The system’s ease of programming, unique DNA cleavage mechanism, and ability to recognize multiple target sites have led to significant advances in the precise and effective editing of a wide range of cellular and organismal genomic loci, and have been widely used in various areas of the life sciences [34]. However, the continuous expression of Cas9 protein leads to off-target effects, which is a major challenge in the application of CRISPR/Cas9 technology. Off-target effects usually occur when the sgRNA fails to precisely match the target DNA sequence, thus introducing additional mutations. The low specificity of Cas9, the imprecision of the sgRNA sequence, and the high concentration of the plasmid in which the sgRNA resides are important factors for off-target effects to occur [35]. Strategies to reduce off-target effects include designing high-specificity sgRNAs, optimizing plasmid concentrations, activating Cas9 repressor expression, and employing transient Cas systems [36] and modifications to produce a new high-fidelity Cas9 (SpCas9-HF1) and a specificity-enhanced Cas9 (eSpCas9) [37,38]. The PAM sequence of the CRISPR/Cas9 system determines its target specificity, and the presence of this sequence in numerous genes allows the CRISPR system to find multiple targets for the correct cleavage of the target gene, thus editing virtually all genes. The PAM sequence varies from strain to strain, and is usually located at the 3′ end of the target DNA [8,27]. For example, the PAM sequences from StCas9 of *Streptococcus thermophilus* and *Meningococcus meningitidis* and SpCas9 of *Streptococcus pyogenes* all show high sequence conservation [39,40]. In the absence of PAM sequences, Cas9 proteins do not cleave target sequences even if the sgRNA is an exact match to the target gene [12]. Thus, the dependence of the PAM sequence imposes a limitation on the target sequence for cleavage. The length of the PAM sequence directly determines the recognition specificity of the CRISPR system, with longer motifs exhibiting enhanced target discrimination capabilities [41]. These properties have important implications for the target-editing capabilities and range of applications of the CRISPR/Cas9 system [27].

## 3. Effects of Gene Editing on Filamentous Fungi

Filamentous fungi pose significant challenges to establishing a mature gene-editing technology platform due to physiological characteristics such as a low HR efficiency, apical growth characteristics, and a scarcity of genetic markers [42,43]. In the process of constructing gene knockout vectors for filamentous fungi, it is usually necessary to go through three rounds of fragment assembly, a procedure that is time-consuming, inefficient, and complicated. The knockout recombinant vector constructed using *pGKO2* as a vector enabled dual screening: negative selection via HSVtK and positive selection via thaumatin resistance (Hyp), markedly enhancing the HR screening efficiency. In some filamentous fungi, the CRISPR system is used for efficient gene knock-in and knock-out. By combining DSBs with HR-driven DNA recombination, it enables the precise disruption of single or multiple genes while facilitating studies on the integration efficiency and copy number [44,45]. When DNA double-strand breaks occur, genomic DNA initiates self-repair mechanisms, which may lead to mutations. The major repair pathways include non-homologous end joining (NHEJ), which may result in base loss, insertion, and substitution at the DNA break point, and the HR pathway, which enables precise editing of the relevant genes with the assistance of foreign donor fragments [46,47,48]. An understanding of these mechanisms is crucial for optimizing gene-editing strategies in filamentous fungi.

Owing to its versatility, CRISPR/Cas9 technology has been widely used in the field of genetic engineering for a variety of research applications, including endogenous gene expression regulation, epigenetic modification, chromosomal locus tagging in living cells, single-stranded RNA editing, and high-throughput gene screening [49,50]. The system’s simplicity allows Cas9 proteins and sgRNAs to be assembled in single or separate vectors, even supporting in vitro construction. Because of the wide distribution of protospacer adjacent motif (PAM) sequences at the target site in the filamentous fungal genome, CRISPR/Cas9 technology can recognize multiple targets, enabling the editing of multiple gene sequences and reducing the use of selective markers, thus improving the efficiency of genome editing. CRISPR/Cas9 gene editing demonstrates significant advantages in genetically engineering both primary and secondary metabolites, enabling their efficient production across diverse filamentous fungi. By demonstrating significant advantages in manipulating diverse filamentous fungi, CRISPR/Cas9 technology has accelerated their use as biofactories for producing numerous metabolites. In particular, in secondary metabolite biosynthesis, CRISPR/Cas9 facilitates the development of bioactive drugs and derivatives while promoting the discovery of novel molecules.

## 4. CRISPR/Cas9 Expression in Filamentous Fungi

### 4.1. Cas9 Protein Expression

The Cas9 protein, as a key component of the CRISPR/Cas9 system, consists of approximately 1400 amino acid residues, assumes the function of a nucleic acid endonuclease, and is capable of site-specific DNA double-stranded breaks through its HNH and RuvC structural domains [51]. With only a single active cleavage structural domain, Cas9 is incapable of achieving the cleavage of double-stranded DNA. The successful expression of the Cas9 gene is significantly influenced by the promoter type, as the transcriptional strength driven by the promoter determines the efficiency of exogenous Cas9 expression; thus, selecting an appropriate promoter is critical for effective CRISPR/Cas9 implementation [8]. In filamentous fungi, most studies have utilized a constitutive promoter that is not induced by external factors to drive the expression of Cas9 proteins, such as *trpC*, *gpdA* and *TEF1* promoters in *A. oryzae*. In addition, promoters such as *xlnA*, *Ham34*, *amyB*, *niiA*, *Otef*, and *hsp70* have also been used for Cas9 expression in filamentous fungi [35]. To minimize off-target effects and maintain Cas9 expression, several inducible promoters such as *PniiA, Pcbh1*, and other modified promoters have also been applied to regulate the expression of Cas9 [52,53,54]. The selection and application of these promoters are important for realizing efficient and specific gene editing by the CRISPR/Cas9 system in filamentous fungi.

The CRISPR/Cas9 system is derived from the adaptive immune system of bacteria and archaea; therefore, its application to fungi necessitates the codon optimization of the genes encoding Cas9 proteins to adapt to the fungal genetic code. In addition, to achieve the nuclear localization of Cas9 in filamentous fungi, a nuclear localization signal (NLS) must be attached to one or both termini, enabling protein translocation to the nucleus via the nuclear import system [8]. The fusion expression of the NLS and Cas9 protein serves as a key indicator of successful Cas9 expression. In filamentous fungi, various nuclear localization sequences have been reported, including the endogenous HTB (derived from the histone H2B), SV40 (from apicomplexan viruses), and VEL (fusion at the Cas9 N-terminal enhancing efficiency) [55,56], and the selection of the appropriate NLS is crucial for the success of the experiment. It has been shown that the classical nuclear localization sequence SV40 NLS functions efficiently in several organisms. The HTB NLS, originating from yeast and evolving in *F. spinosum*, efficiently localizes Cas9 in filamentous fungi without compromising its activity [56,57]. In addition, the integration of the enhanced green fluorescent protein (eGFP) gene into the optimized Cas9 gene can be used to verify the expression of the Cas9 protein in the recipient fungi and the intensity of its expression by fluorescence assay [35]. The implementation of these strategies provides important technical support for the effective application of the CRISPR/Cas9 system in filamentous fungi.

### 4.2. Expression of sgRNA

In the CRISPR/Cas9 system, guide RNA (sgRNA) plays a crucial role, and its transcription is usually driven by the RNA polymerase type III *U6* promoter, which has been widely adopted because of its highly efficient transcriptional activity [51]. In the genetic manipulation of filamentous fungi, the *U6* promoter-mediated sgRNA transcription of the CRISPR/Cas9 system has become a mainstream method. Studies have shown that the efficiency of single promoter-driven sgRNA expression is superior to that of dual promoter systems [35]. It has been shown that the *SNR52* promoter in *Saccharomyces cerevisiae* efficiently transcribes precursor sgRNAs containing leader sequences, which are processed into functional sgRNAs [35]. In addition, this promoter can also activate single-guide RNA (sgRNA) transcription in *A. fumigatus* and *Pulsatilla vulgaris* [51]. In filamentous fungi, the abundance of tRNA genes provides a variety of RNA polymerase type III promoters that activate sgRNA expression, thereby facilitating the editing of multiple genes. Some of the tRNA promoters, such as *ptRNAGlyGCC*, *ptRNALeuTAA*, *ptRNATyrGTA*, and *ptRNAGlyTCC*, show higher transcriptional efficiencies than the *U6* promoter in filamentous fungi [58,59,60]. However, tRNA promoters exhibit a limited regulatory capacity, and therefore their expression efficiency in vitro and in vivo has been intensively investigated for efficient sgRNA expression [8]. Plasmids containing AMA1 sequences from *A. oryzae* are often used for the co-expression of Cas9 and sgRNAs, which support plasmid-autonomous replication [61]. These studies provide important molecular tools and strategies for optimizing the CRISPR/Cas9 system in filamentous fungi.

A novel CRISPR/Cas9 system based on the 5S rRNA gene promoter was developed. The system significantly enhanced the transformation efficiency, successfully generating tens of transformants. With a short homologous donor DNA fragment (40 bp), it achieved 100% precise gene modification rates [62]. This CRISPR/Cas9 system has been applied to fungal chromosome engineering, enabling multiple gene insertions and large-scale DNA deletions. The technology is particularly suitable for chromosome design in *A. niger*, enabling high-throughput, large-scale genome engineering to enhance the gene editing efficiency [63]. This advancement provides an efficient new tool for fungal genome editing.

## 5. Transformation of Cas9 Protein and sgRNA in Filamentous Fungi

**Figure 2:**
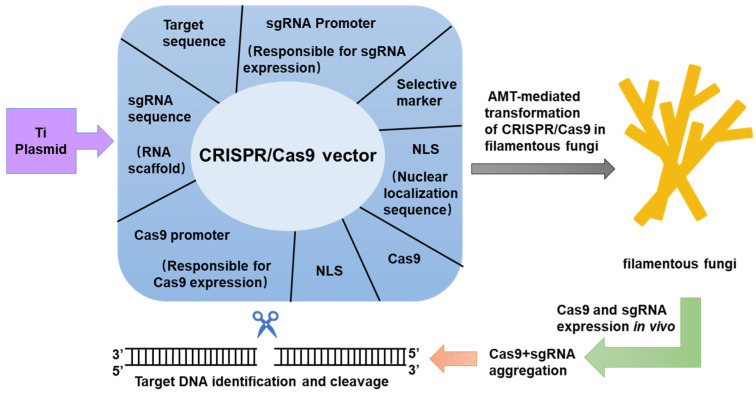
CRISPR/Cas9 is responsible for successful genome editing in filamentous fungi. The Agrobacterium-mediated transformation of CRISPR/Cas9 is delivered via Ti plasmids in filamentous fungi. A general CRISPR/Cas9 vector contains the Cas9 gene, gRNA sequence, promoters for Cas9 and gRNA expression, a target sequence, a selective marker, and a nucleotide signal sequence known as NLS. CRISPR/Cas9 expression occurs in vivo, aggregating a large number of Cas9 and sgRNA molecules. The sgRNA directs the Cas9 protein to the target sequence, which recognizes the protospacer adjacent motif on the template strand. The Cas9 nuclease binds to specific genomic loci in the fungal genome. The HNH domain of the Cas9 protein cleaves the complementary target strand, inducing double-strand breaks.

In fungal genome editing, the application of the CRISPR/Cas9 system involves the delivery of the Cas9-sgRNA-CRISPR complex into fungal cells via host-specific vectors. The pre-assembled CRISPR/Cas9 system can be delivered directly to the target cell when the Cas9 ribonucleoprotein (RNP) complex is present, and this method is particularly effective for transient gene expression. In the absence of markers, vectors based on AMA1, a sequence that is autonomously maintained in Aspergillus, have been shown to be extremely effective for gene editing in filamentous fungi [64,65]. In terms of protein expression, the sequences of Cas9 and sgRNA can be co-localized for expression in a single vector or in two different vectors, respectively (Figure 2). A 2016 study compared the results of two different expression methods in *A. fumigatus* and showed that the efficiency and accuracy of the single-vector expression system was significantly better than that of the dual-vector expression system [35]. Numerous studies have further confirmed that first stably transforming Cas9-expressing genes into recipient cells, screening for positive strains expressing Cas9 proteins, and subsequently transfecting in vitro-transcribed sgRNAs into Cas9-positive cells for gene editing using in vitro-transcribed sgRNAs is a more effective strategy [66,67]. In addition, plasmid-free systems allow in the vitro synthesis of Cas9 and sgRNA, the formation of stable RNP complexes, and the introduction of these complexes into cells for gene editing by electroporation or protoplast transformation (PMT) techniques [8]. The development of these approaches has provided diverse technological pathways for fungal genome editing.

Polyethylene glycol (PEG)-mediated transformation and Agrobacterium-mediated transformation (AMT) are two widely used techniques for delivering the CRISPR/Cas9 system into fungal cells [35]. Given the simplicity of PEG-mediated transformation in fungi, numerous studies have favored this method for introducing Cas9 proteins and guiding RNAs (gRNAs) into fungi [68]. Additionally, it has been demonstrated that AMT is also capable of efficient genome editing in fungi [69]. Collectively, these transformation methods provide versatile tools for fungal genome editing.

## 6. Regulation of the HR/NHEJ Pathway in Filamentous Fungi

**Figure 3:**
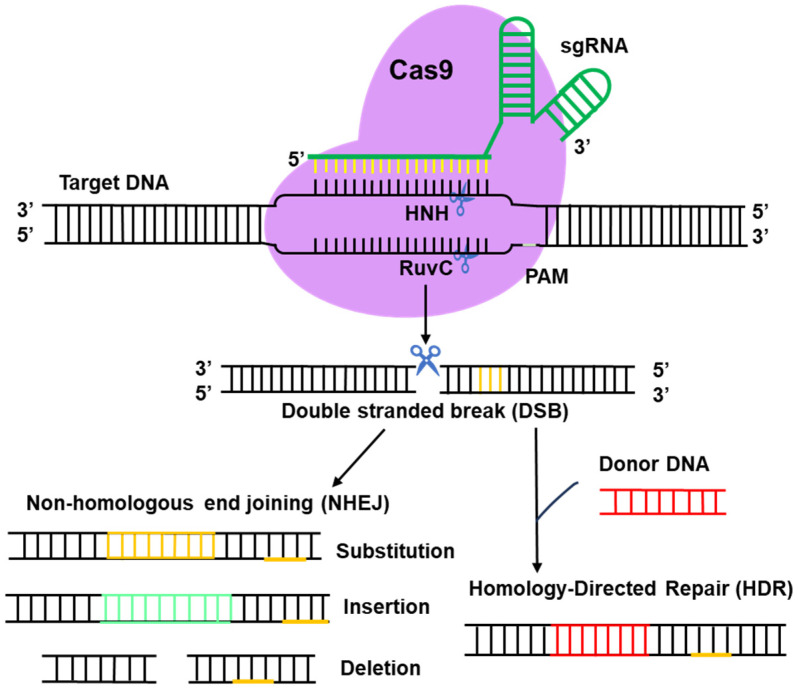
Schematic diagram of the CRISPR–Cas 9 system molecular mechanism. The CRISPR/Cas 9 system is composed of Cas9 and sgRNA. The Cas9 protein contains two nuclease domains—the HNH domain, which cleaves complementary DNA strands, and the RuvC domain, which cleaves non-complementary DNA strands. The sgRNA binds to the Cas9 protein to form the CRISPR/Cas9-sgRNA complex to edit the genome sequences. The Cas9–sgRNA complex unwinds the dsDNA, and the complementary sequence in sgRNA anneals to one of DNA strands. Upon binding, the endonuclease domains cleave both DNA strands three bases upstream of the protospacer-adjacent motif (PAM) sequence. The double-strand break (DSB) in DNA forms and then is repaired either by non-homologous end joining (NHEJ) or by a homology-directed repair (HDR) pathway, if an appropriate donor is present. Imprecise NHEJ-mediated repair can produce insertion/deletion/substitution of variable lengths at DSB sites. HDR-mediated repair can introduce precise point mutations at the target site through substitutions, insertions, or deletions, depending on the donor DNA template.

Genome-editing technology is a key tool used in genetics research to precisely modify genomes. In filamentous fungi, the CRISPR/Cas9 system triggers DNA double-strand breaks (DSBs) through the interaction of the HNH structural domain of Cas9 nuclease with complementary target sequences. Subsequently, the activated gene repair mechanism takes different repair pathways depending on the presence or absence of a donor template: in the absence of a donor template, random nucleotide insertions, substitutions, or deletions are generated through the non-homologous end joining (NHEJ) pathway, whereas in the presence of a donor template, HR or HDR is used to achieve the modification, replacement, deletion, or precise insertion [10] (Figure 3). Short homology arms at both ends of the donor template DNA enable the insertion or deletion of DNA fragments during microbial-mediated end-joining repair. These repair mechanisms have been applied in fungal genome editing, allowing the induction of multiple types of mutations at the target genomic locus [9]. The key to efficient genome editing lies in the induction of DSBs near the target genomic locus. These breaks significantly enhance the probability of DNA substitution through HR, and may also trigger the error-prone NHEJ process [70,71]. The development of these techniques has provided powerful tools for precise fungal genome editing.

The cell cycle has a decisive influence on the selection of DNA repair pathways. HR occurs predominantly in late S-phase to G2, whereas nonhomologous end-joining (NHEJ) operates mainly in G1 but remains active throughout the cell cycle [72,73]. HR, as a high-fidelity DNA repair mechanism, relies on homologous templates to guide the DNA repair process. The probability of HR occurring during DNA damage repair is significantly increased in the presence of homologous DNA fragments [35]. In contrast, the NHEJ repair pathway repairs double-strand breaks by directly ligating DNA ends. The Ku70 and Ku80 proteins are essential for this process, but it often introduces sequence alterations. Deleting the *Ku70* and/or *Ku80* genes in *Neurospora crassa* increased the frequency of gene replacement from 10–30% to 100%. Deleting the *ku70* and *ku80* genes in the NHEJ pathway increased the HR frequency in filamentous fungi, thereby enhancing the genome editing precision [74].

Advances in transformation technologies and the development of selective markers offer the possibility of HR in many filamentous fungal species. The efficiency of genetic engineering has been significantly enhanced by generating mutants with deletions in genes associated with nonhomologous end joining (NHEJ), such as *YKU70-YKU80* and *ligD*. The NHEJ repair process requires at least 10 genes, including *YKU70*, *YKU80*, *DNL4*, *LIF1*, *SIR2*, *SIR3*, *SIR4*, *RAD50*, *MRE11*, and *XRS2*, most of which are essential for in vivo gene editing [75]. Some of the NHEJ mutants exhibit aberrant processing and the reattachment of double-strand breaks. In addition to *DNL4* and *LIF1*, all other NHEJ genes are required for maintaining stable telomeric repeat sequences. Proteins in the NHEJ pathway interact with linear DNA ends through direct or indirect binding to other components [75]. These findings provide an important molecular basis for understanding and manipulating DNA repair mechanisms in filamentous fungi.

## 7. Application of CRISPR/Cas9 Technology in Filamentous Fungi

In recent years, CRISPR/Cas9 genome editing technology has triggered a revolution in the field of filamentous fungi research. The technique has been successfully adapted for *A. oryzae* and subsequently extended to *A. vulgaris* and *A. conidiosus* [76]. The precise gene editing of filamentous fungi using CRISPR/Cas9 has established novel pathways for developing highly efficient strains [8]. So far, this gene-editing tool has been used in the genome editing of a variety of filamentous fungi, demonstrating its importance for enhancing strains related to agriculture, industry, and pharmaceuticals.

### 7.1. Application of CRISPR/Cas9 in Engineered Strains of Filamentous Fungi

CRISPR/Cas9 has significantly advanced the engineering of engineered filamentous fungal strains, enabling targeted improvements for applications in industrial biotechnology and pharmaceutical development.

In enhancing secondary metabolite production, CRISPR/Cas9-mediated gene editing has enabled the optimization of fungal strains for high-value metabolite biosynthesis. For instance, in *Monascus pilosus*, the targeted knockout of the DNA damage response gene *mpclr4* via CRISPR/Cas9 increased the Monacolin K yield by 52.6% under controlled fermentation conditions, demonstrating its potential for lipid-lowering drug synthesis [77]. Similarly, CRISPR/Cas9-edited strains of *A. Niger*, where competitive metabolic pathways were disrupted, showed an increased production of citric acid, an essential feedstock for manufacturing processes [35].

In terms of industrial enzyme optimization, engineered strains are pivotal for their production. CRISPR/Cas9 has been used to enhance cellulase and xylanase secretion in *Trichoderma reesei* by targeting transcriptional repressors [78]. In *Ashbya gossypii*, CRISPR-mediated *ADE2* knockout improved vitamin B2 titers by 38% while eliminating off-target mutations, highlighting its precision in metabolic engineering applications [79].

Regarding stress tolerance and process scalability, *mplig4* is associated with the DNA damage response, and after editing with the CRISPR/Cas9 system, studies demonstrated that the inactivation of *mplig4* blocked the NHEJ pathway, resulting in a significantly reduced tolerance to DNA damage agents [77]. This engineered DNA repair pathway enhances fungal tolerance to reactive oxygen species during fermentation and improves bioreactor performance.

In summary, these applications highlight CRISPR/Cas9′s transformative role in tailoring filamentous fungi for bioproduction, therapeutic development, and industrial scalability.

### 7.2. Application of CRISPR/Cas9 Technology to Editing Filamentous Fungi in Agriculture

Numerous crops are cultivated to provide essential resources for human societies, including food and condiments. However, genetic studies of filamentous fungi that cause disease in cash crops have been hindered by the lack of precise targeted mutagenesis and gene replacement techniques. The introduction of CRISPR/Cas9 technology has revolutionized the study and management of these plant pathogens.

The targeted knockdown of two genes, *USTA* and *UvSLT2G*, in the rice blast fungus *Magnaporthe oryzae* was achieved using the RNA polymerase type III promoter-driven CRISPR/Cas9 system [59]. A set of CRISPR/Cas9 genome-editing technological processes, including the target sequence design, was detailed by Arazoe et al., including CRISPR/Cas9 expression vector construction, and the realization of precise editing transformation in the genome of *Phytophthora ramorum*, which is capable of producing effective targeted gene disruption, base editing, and reporter gene knock-in without the additional modification of host genes [9]. In *Phytophthora capsici* (*pepper blight*), the application of CRISPR/Cas9 genome-editing technology through the *RPL41* promoter and codon-optimized Cas9 was transformed in a protoplast-mediated manner to achieve gene disruption via the NHEJ pathway. It was found that the deletion of *ΔN837* conferred resistance to inhibitors of oxysterol-binding protein homologs in the soybean yellow rot fungus *Phytophthora sojae*, and these pure mutants exhibited adaptations similar to those of the wild type, offering the possibility of developing novel oxysterol-binding protein homolog inhibitors and fungicides [80]. As an important pathogen of soybean, rapid and efficient genome editing of the HR or NHEJ pathway of *Phytophthora sojae* via CRISPR/Cas9, using a constructed *RPL41* promoter and codon-optimized Cas9, successfully disrupted single genes. By targeting the RXLR effector gene *Avr4/6*, it was found that in the absence of a homologous template, the NHEJ-mediated repair of NHEJ-induced Cas9-induced double-stranded breaks in soybean DNA resulted in an insertion deletion; however, in the presence of donor DNA, HDR occurred, leading to the replacement of *Avr4/6* by the *NPT II* gene. The application of CRISPR/Cas9 technology in these studies provides a powerful tool for functional genomics studies of epidemics and offers new methods for the more effective control of pathogens [81].

### 7.3. CRISPR/Cas9 Applied to Industrial Strains of Filamentous Fungi

Metabolites produced by filamentous fungi play a key role in industry, particularly in manufacturing processes such as detergents, food, paper, textiles and enzymes, as well as in food fermentation and the recombinant expression of therapeutic proteins. However, the production capacity of wild-type strains can no longer meet the growing industrial demand. To bridge this gap, the genetic improvement of these strains to enhance their biomanufacturing efficiency has become a strategic priority in the field of biotechnology.

The application of CRISPR/Cas9 genome editing technology in filamentous fungi began with the study of *T. reesei*, a strain that has been widely studied for its importance in the production of lignocellulolytic enzymes and heterologous proteins. In recent years, the efficient gene editing of *T. reesei*, including gene insertion and deletion, has been achieved through the optimization of the Cas9 gene and the knockout of *SxlR*-related genes, resulting in the enhanced production of lipase and expression of heterologous proteins, as well as the removal of nonessential genes [82,83]. The industrial strain of *Streptomyces penicillinaris* (*Acremonium chrysogenum*), used as a commercial producer of cephalosporin C, achieved the simultaneous targeting of dual motifs through an improved CRISPR/Cas9 technology based on a chimeric promoter construct of *U6*/tRNA, combined with the use of HDR template donor DNA. The system successfully and efficiently deleted a long DNA fragment, significantly enhancing the yield of cephalosporin C produced by *Streptomyces penicillinaris* [84]. *A. oryzae*, an industrial strain widely used for the expression of enzymes and heterologous proteins, was enhanced by the CRISPR/Cas9 technique in combination with a plasmid containing an AMA1 auto-replicating sequence for Cas9 and sgRNA expression. Additionally, the modification of nonessential genes by single base pair insertion or deletion further increased the production of targeted heterologous proteins [85,86]. Using the CRISPR system for gene replacement, the genome of the model organism *Neurospora crassa* was effectively modified to achieve efficient cellulase expression by knocking in the *clr-2* gene driven by the β-tubulin promoter [87]. *Penicillium subrubescens*, as an ascomycete capable of degrading sugars, is considered a potential industrial cellular factory for enzyme production. Its low HR efficiency during DNA repair limits its genetic manipulation possibilities. Knockdown of the *ku70* gene, which is responsible for non-homologous end-joining, by the CRISPR/Cas9 system successfully produced a *Δku70* mutant strain with high HR efficiency [88]. These research advances demonstrate the great potential of CRISPR/Cas9 technology in the genome editing of filamentous fungi and provide a new strategy for strain improvement in industrial production.

### 7.4. CRISPR/Cas9 Applied to Filamentous Fungi Strains for the Pharmaceutical Industry

Secondary metabolites produced by filamentous fungi, such as paclitaxel, have become clinically important therapeutic agents. Paclitaxel, as an important diterpenoid, is primarily used in anticancer therapy [89]. El-Sayed and his team employed the CRISPR/Cas9 system to block the sterol metabolism pathway in filamentous fungi by knocking out squalene synthase and quinoa phytosterol synthase, thereby increasing paclitaxel production [90]. The industrial fungus *Ashbya gossypii*, renowned for its ability to produce vitamin B2, knocked down the *ADE2* gene by CRISPR/Cas9 technology and transferred the vector, and PCR characterization and DNA sequencing confirmed that the system was able to eliminate the adverse effects that arose after multiple rounds of manipulation, thus enhancing the use of the strain [35]. In the higher fungus *Ganoderma lucidum*, knocking out the *URA3* gene via CRISPR/Cas9 technology has emerged as a highly effective metabolic engineering strategy. Specifically, codon-optimized Cas9 and in vitro-transcribed sgRNA can accurately introduce DNA double-strand breaks, disrupting *URA3* gene function via NHEJ mechanisms. Inactivating this gene blocks the pyrimidine nucleotide synthesis pathway, forcing cells to enhance the biosynthesis of triterpenoids such as ganoderic acid through metabolic reprogramming. Ganoderic acid is a class of secondary metabolite with hypotensive, hypolipidemic and hepatoprotective activities. Its increased production provides potential resources for the development of adjuvant therapy for liver diseases [91]. These studies indicate that the application of CRISPR/Cas9 technology in filamentous fungi provides a new strategy for industrial production and drug development.

### 7.5. CRISPR/Cas9 Applied to Other Strains of Filamentous Fungi

As an efficient genome-editing tool, the CRISPR/Cas9 system has demonstrated significant application value in the field of fungal biology, particularly in elucidating the molecular interactions between fungal pathogens and their hosts. With the continuous optimization and improvement of this technology, its application is poised to expand into the field of genetic diagnosis, propel the development of protein engineering, enhance the specificity and catalytic efficiency of enzymes, and pave new pathways for the development of new drugs. In addition, the multi-genome editing capability of the CRISPR/Cas9 system enables the simultaneous modification of multiple metabolic enzymes in the byproduct production pathway, potentially promoting the accumulation of target products or diminishing deleterious metabolites in filamentous fungi [51]. Therefore, the application of the CRISPR/Cas9 system in filamentous fungi ushers in a new era of innovation and development in this field.

Within the research and application domain of filamentous fungi, notable variations exist in experimental approaches and gene-editing efficiencies when applying CRISPR/Cas9 technology across different strains (Table 1). However, CRISPR/Cas9 technology remains a pivotal tool.

## 8. Conclusions and Outlook

CRISPR/Cas9 technology has emerged as a groundbreaking tool for filamentous fungal research, surmounting long-standing hurdles caused by the unique biology of filamentous fungi, such as their limited genetic markers, low heterokaryon division efficiency and low efficiency of HR. In this review, we comprehensively explore the progress and application of CRISPR/Cas9 in fungal research, highlighting its versatility in achieving precise gene knock-in, knock-out, and multi-site modification, thereby significantly advancing the research of fungal functional genomics and metabolic engineering. By optimizing an optimized delivery system, enhancing promoter-driven Cas9 expression, and improving the sgRNA design, the editing efficiency is significantly improved while off-target effects are minimized—a pivotal milestone for basic research and practical applications. The integration of CRISPR/Cas9 with fungus-specific repair pathways, particularly the regulation of HR and NHEJ mechanisms, has enabled unprecedented precision in genome engineering. Case studies of different fungal species further confirm the technology’s crucial role in enhancing secondary metabolite diversity, optimizing industrial enzyme expression, and developing anti-fungal drug target screening, marking a transformative shift in filamentous fungal genome engineering from basic research to industrial-pharmaceutical hybrid applications. These successes underscore CRISPR/Cas9′s role as a cornerstone tool for strain improvement, enabling modifications to meet industrial, agricultural, pharmaceutical, and ecological demands.

Despite these achievements, challenges persist. Strain-specific variabilities in PAM sequence limitation, editing efficiency, and residual off-target effects necessitate continued innovation through tool iterations and cross-disciplinary breakthroughs. In the future, CRISPR/Cas9-based filamentous fungi gene-editing technology can overcome the bottlenecks associated with the transformation efficiency and editing precision through delivery system innovation, the diversified upgrading of technical tools and the precise regulation of repair mechanisms. At the same time, the integration of multi-omics analysis and machine learning algorithms can optimize metabolic pathway design, drive applications in synthetic biology in industrial enzyme production or drug synthesis, and target the design of filamentous fungal cell factories to achieve the intelligent biosynthesis of high value-added products. Advanced transient expression systems and plasmid-free RNP delivery can further enhance security and scalability. Combined with metabolic pathway engineering, the industrial potential of filamentous fungi can be unlocked. In addition, it is essential to establish a standardized editing process and enhance the biosafety specifications of gene-edited strains, thereby balancing technological innovation with ethical risks, and ultimately maximizing the technology’s impact in the fields of basic research, biological manufacturing, green agriculture and precision medicine development.

In conclusion, CRISPR/Cas9 technology holds great promise for advancing our understanding and utilization of filamentous fungi. By integrating basic research with practical applications, this technology drives innovation in agriculture, biomanufacturing and medicine, establishing its indispensable role in the era of precision genome engineering.

## Figures and Tables

**Table 1:** An overview of the application of the CRISPR/Cas9 system in filamentous fungi.

Strain Name	Experimental Strategy	Editing Efficiency	Major Findings	References
*A. oryzae*	Cas9 (*amyB* promoter), sgRNA (*U6* promoter), NHEJ repair	10–20%	CRISPR/Cas9-mediated genome editing was achieved for the first time in *A. oryzae*, demonstrating the feasibility of the system.	[92]
*Glarea lozoyensis*	Cas9 (*trpC* promoter), sgRNA (*U6* promoter), NHEJ repair	Approximately 80%	CRISPR/Cas9-based gene editing tool is efficient for manipulating genes in *G. lozoyensis*	[93]
*Myceliophthora thermophila*	Cas9 (*Ptef1* promoter), sgRNA (*U6* promoter), HR repair	Approximately 100%	Characterized a novel regulator MtTRC-1 in *M. thermophila*, which regulated cellulase production through direct transcriptional regulation of the *Mthac-1* and *Mtcbh-1* genes.	[94]
*A. fumigatus*	Cas9 (*tef1* promoter), sgRNA (*gpdA* promoter), HR repair	Approximately 10%	A CRISPR/Cas9-mediated gene-editing strategy for improving the endoglucanase activity of *A. fumigatus* LMB-35Aa strain was successfully used, which constitutes the first report of heterologous cellulase production in filamentous fungi using this technology.	[95]
*A. nidulans*	Cas9 (*PgpdA* promoter), sgRNA *(T7* promoter), MMEJ repair	Approximately 100%	The production of L-malic acid was enhanced by approximately 9.6 times.	[96]
*F. fujikuroi*	Cas9 (*Ptef1* promoter), sgRNA (*5S* rRNA promoter), HR repair	dual: 25% 75% triple: 12.5~37.5%	The production of GA3 was enhanced by approximately 50.19%.	[97]
*T. reesei*	Cas9 (*qai5* promoter), sgRNA (*T7* promoter), HR repair	46.7%	The production yield of ilicicolin H reached 4.8 mg/L.	[98]
*Berkleasmium sp. Dzf12*	Cas9 (*Ptef1* promoter), sgRNA (*U6* snRNA promoter), HR repair	16.6%~50%	DHN and spirobisnaphthalenes were found to have a biosynthetic relationship.	[99]
*M. ruber*	Cas9 (*PgpdA* promoter), sgRNA (*T7* promoter), NHEJ repair	18.2%	MpigI and MpigI’ were directly related in the production of Monascus pigments.	[100]
*Cordyceps militaris*	Cas9 (*Pcmgpd* promoter), sgRNA (*Ptrpc* promoter), NHEJ repair	17.9%	Protein modification and promoter strength evaluation were performed, along with the deletion of 10 kb biosynthetic clusters.	[101]

## Data Availability

No new data were created or analyzed in this study. Data sharing is not applicable to this article.
